# 2432: A close examination of anti-retroviral drug selection and management in the optima study

**DOI:** 10.1017/cts.2017.137

**Published:** 2018-05-10

**Authors:** Yuan Huang, Sheldon T. Brown, Shuangge Ma, Tassos Kyriakides

**Affiliations:** ^1^Yale School of Medicine, Guilford, CT, USA

## Abstract

OBJECTIVES/SPECIFIC AIMS: Effective HIV therapeutic options for persons with advanced HIV disease whose regimens have failed multiple times are limited. Current clinical practice utilizes regimens comprised of combinations of anti-retroviral (ARV) drugs. Despite the widespread use of ARV medications, optimization of initial treatment composition and subsequent management remains challenging. The goals of this study are (a) to better understand the ARV treatment structuring using prior clinical and patient information including virtual phenotype data and measures of viral load and CD4 cell count. We evaluated the potential impact of ARV strategies on AIDS-defining events and mortality; (b) to assess and understand differences of treatment composition and management when comparing standard ARV strategy (<5 ARVs) with an intensive ARV strategy (at least 5 ARVs). METHODS/STUDY POPULATION: OPTIMA was a tri-national (United States, Canada, and United Kingdom) randomized open label of alternative ARV treatment strategies for patients with advanced HIV disease (CD4≤300 cells/mm^3^) and evidence of resistance to 3 classes of ARV medications. OPTIMA used a 2×2 factorial design where the 2 factors were an ARV-free period Versus not; and standard Versus intensive ARV regimen. In this study, we focus on participants enrolled in OPTIMA at US participating sites and utilize demographic and clinical data including baseline virtual phenotype, ARV-related data (initial assignments and changes with drugs and dosages), follow-up lab data, AIDS-defining events, and vital status. RESULTS/ANTICIPATED RESULTS: Among 278 US-OPTIMA participants, 146 were randomly assigned to the standard ARV strategy and the rest were assigned to the intensive ARV strategy. Although not the sole factor, baseline virtual phenotype was used in selecting ARV medications within each assigned strategy. Participants in the standard arm exhibited better agreement between virtual phenotype results and the individual drugs selected for their regimen compared with participants in the intensive arm. This agreement had an almost statistically significant impact on survival time. No significant difference was detected in the frequency of ARV changes between standard and intensive ARV groups. DISCUSSION/SIGNIFICANCE OF IMPACT: Even though per design, OPTIMA assigned participants to an ARV strategy using a binary factor (standard vs. intensive ARV) and assessed its effect on HIV-related disease at a coarse level, the trial’s design and rich database allowed for a closer examination of the ARV drug initial selection and subsequent management. Our findings summarize the patterns and discuss the effects of ARV and their management, on AIDS-defining events and survival. Such findings could provide preliminary, yet important insight, in understanding ARV use practice and could inform the conduct of future HIV treatment trials. Since the trial’s randomization was at the ARV strategy level and not the individual ARV drugs, findings cannot be described in terms of causal pathways for specific ARVs.

